# Horizontal Transmission of Intracellular Insect Symbionts via Plants

**DOI:** 10.3389/fmicb.2017.02237

**Published:** 2017-11-28

**Authors:** Ewa Chrostek, Kirsten Pelz-Stelinski, Gregory D. D. Hurst, Grant L. Hughes

**Affiliations:** ^1^Department of Vector Biology, Max Planck Institute for Infection Biology, Berlin, Germany; ^2^Department of Entomology and Nematology, University of Florida, Gainesville, FL, United States; ^3^Institute of Integrative Biology, University of Liverpool, Liverpool, United Kingdom; ^4^Department of Pathology, Institute for Human Infections and Immunity, Center for Biodefense and Emerging Infectious Disease, Center for Tropical Diseases, University of Texas Medical Branch, Galveston, TX, United States

**Keywords:** horizontal transmission, plant-mediated transmission, host-switching, plant-symbiont interaction, endosymbiont, *Wolbachia*, *Rickettsia*, Cardinium

## Abstract

Experimental evidence is accumulating that endosymbionts of phytophagous insects may transmit horizontally via plants. Intracellular symbionts known for manipulating insect reproduction and altering fitness (*Rickettsia, Cardinium, Wolbachia*, and bacterial parasite of the leafhopper *Euscelidius variegatus*) have been found to travel from infected insects into plants. Other insects, either of the same or different species can acquire the symbiont from the plant through feeding, and in some cases transfer it to their progeny. These reports prompt many questions regarding how intracellular insect symbionts are delivered to plants and how they affect them. Are symbionts passively transported along the insect-plant-insect path, or do they actively participate in the process? How widespread are these interactions? How does symbiont presence influence the plant? And what conditions are required for the new infection to establish in an insect? From an ecological, evolutionary, and applied perspective, this mode of horizontal transmission could have profound implications if occurring frequently enough or if new stable symbiont infections are established. Transmission of symbionts through plants likely represents an underappreciated means of infection, both in terms of symbiont epidemiology and the movement of symbionts to new host species.

## Introduction

Bacteria–insect symbioses, ranging from mutualistic to parasitic interactions, are pervasive in nature. Their commonness is likely due to the profound influence of the symbionts on host biology, whereby they can be required for hosts’ survival, increase host fitness, or manipulate reproduction to promote their own spread (Moran et al., 2008). Insect–symbiont associations can either be obligate or facultative for the host, symbionts can have intracellular or extracellular lifestyles, and they can be transmitted vertically, horizontally, or by the combination of both strategies (Moran et al., 2008; Ebert, 2013).

While vertical transmission is the most frequent transmission mode for facultative intracellular symbionts of insects, incongruence between the host and symbiont phylogenies indicates that many horizontal symbiont transfers have occurred over evolutionary time. These events are important, enabling symbionts to extend their host range and for the hosts, which, together with the new symbiont, gain new adaptations (Sudakaran et al., 2017). Most of the directly observed horizontal symbiont transfer events involve parasitoids, as is the case for *Arsenophonus*-uninfected parasitoids acquiring the infection while developing within the same host with the infected counterparts (Duron et al., 2010). Parasitoids may also serve as phoretic vectors, spreading *Wolbachia* from infected to uninfected whiteflies (Ahmed et al., 2015) and *Hamiltonella defensa* and *Regiella insecticola* by sequentially stabbing infected and uninfected aphids (Gehrer and Vorburger, 2012). Finally, parasitoids may acquire infection while developing inside an infected host (Heath et al., 1999; Chiel et al., 2009). In addition to these routes, intracellular insect symbionts can occasionally spread through the insects’ food sources, such as in the cases of predation and cannibalism of symbiont-harboring individuals, which has been demonstrated in isopods (Le Clec’h et al., 2013). Transmission via food has also been seen in aphids feeding on a common artificial diet (Darby and Douglas, 2003). However, in general, this form of transmission may be more common for gut compared to intracellular symbionts.

**FIGURE 1:**
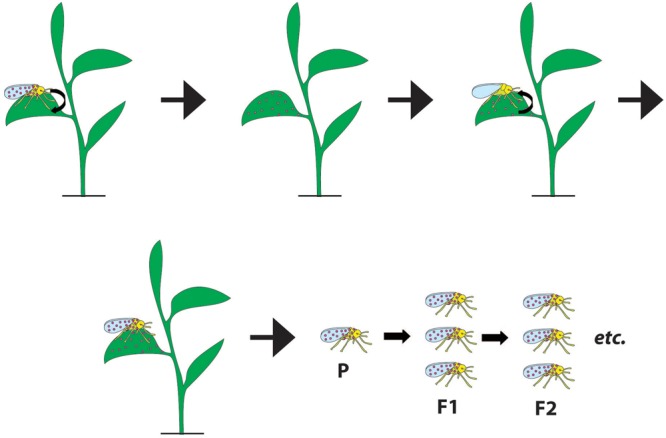
Schematic overview of the plant-mediated symbiont transfer. The process requires insect-to-plant symbiont transfer, symbiont’s survival, and persistence in a plant (where the symbiont may or may not disseminate), plant-to-insect transfer and successful establishment in the new insect. A subsequent vertical transmission of an acquired symbiont can increase its prevalence, however, even the transient infections could impact host populations. Small black arrows indicate symbiont moving between organisms. P stands for the parent, F1 is the progeny of the parent, and F2 is the progeny of the F1.

There has been increasing interest in the role of symbiont transmission through plants. In the case of herbivores, indirect evidence for horizontal transmission via plant diet comes from the observations that insects that feed upon common plants possess similar symbionts (Sintupachee et al., 2006; Stahlhut et al., 2010; Morrow et al., 2014). In this review, we examine the mechanistic process involved in symbiont transmission through the plant, first detailing the experimental systems studied to date, before examining four phases of transfer: from insect to plant; establishment or residence in the plant; uptake of a symbiont by an insect feeding upon that plant and then transmission of the symbiont by the insect to its progeny (**Figure 1**). We then discuss the ecological and evolutionary significance of plant-mediated transmission of symbionts before outlining areas for future research.

## Experimental Systems In Plant-Mediated Symbiont Transfer Studies

Intracellular bacteria usually regarded as insect symbionts, such as *Rickettsia, Wolbachia, Cardinium*, and bacterial parasite of the leafhopper *Euscelidius variegatus* (BEV), have been shown to pass from herbivorous insects to plants, localize to plant tissues, and infect or contaminate the naïve insects feeding on the plant (Purcell et al., 1994; Caspi-Fluger et al., 2012; Gonella et al., 2015; Li et al., 2017). All four symbionts are primarily vertically transmitted, they possess several characteristics ensuring success within the insect host and in the instances discussed here they were associated with phloem feeding insects (**Table 1**).

**Table 1:** Summary of the literature on experimental plant-mediated horizontal symbiont transfer.

Symbiont	Symbiont	Plant	Symbiont	Type of evidence for	Reference
	donor		recipient	transfer to the recipient	
BEV	Leafhopper *Euscelidius variegatus*	Rye grass *Lolium perennae*, barley *Hordeum vulgare*, sugar beet *Beta vulgaris*	Leafhopper *Euscelidius variegatus*	CFUs determination	Purcell et al., 1994
*Rickettsia*	Whitefly *Bemisia tabaci*	Cotton *Gossypium hirsutum*	Whitefly *Bemisia tabaci*	PCR	Caspi-Fluger et al., 2012
*Cardinium*	Leafhopper *Scaphoideus titanus*	Grapevine *Vitis viniera*	Leafhoppers *Macrosteles quadripunctulatus* and *Empoasca vitis*	qPCR or FISH	Gonella et al., 2015
*Wolbachia*	Whitefly *Bemisia tabaci*	Cotton *Gossypium hirsutum*	Whitefly *Bemisia tabaci*	PCR, qPCR, vertical transmission test	Li et al., 2017

Horizontal transfers of these symbionts are important as these organisms can profoundly influence their hosts. *Rickettsia*, an alphaproteobacterium, increases heat tolerance (Brumin et al., 2011), fitness (Himler et al., 2011), as well as susceptibility to insecticides (Kontsedalov et al., 2008) of the whitefly *Bemisia tabaci*. Notably, this *Rickettsia* is closely related to the plant-pathogenic *Rickettsia* causing Papaya bunchy top disease, vectored by leafhoppers *Empoasca papayae* (Davis et al., 1998; Weinert et al., 2009). Thus, *Rickettsia* could easily transition between being an insect mutualist and a plant pathogen. *Wolbachia*, closely related to *Rickettsia*, is a symbiont well known for manipulating insect reproduction (Werren et al., 2008), for protecting insect hosts from pathogens (Hedges et al., 2008; Teixeira et al., 2008), and has also been suggested to increase the fitness of the whiteflies (Xue et al., 2012). *Cardinium*, a member of the *Bacteroidetes* clade, manipulates reproduction, immunity, and fitness of various insects (Hunter and Zchori-Fein, 2006). Finally, BEV, a gammaproteobacterial symbiont of the leafhopper *E. variegatus*, is pathogenic: it reduces fecundity, longevity and increases leafhopper’s developmental time (Purcell et al., 1986; Purcell and Suslow, 1987), but also decreases X-disease transmission by leafhopper to the celery plants (Purcell and Suslow, 1987). From the bacteria discussed here, it is also the only one that can be cultivated outside of host cells (Purcell et al., 1986).

### Symbiont Passage from Insect to Plant

Plant-mediated horizontal transmission initially requires for a symbiont to be passed from an insect to a plant. The route of symbiont transmission to a plant may differ depending on the transmitting insect’s anatomy, physiology, and the mechanism of feeding. Symbiont factors are likely equally important, including symbiont density and tissue distribution (Caspi-Fluger et al., 2012). It has been noted that symbionts exhibiting a scattered infection pattern, whereby bacteria reside in the bacteriome, hemolymph, and other organs, are more likely to be horizontally transmitted than the ones restricted to bacteriome and ovaries (Caspi-Fluger et al., 2012). While *Wolbachia* in *B. tabaci* can exhibit both infection patterns (Ahmed et al., 2015), insects with the scattered infection transferred symbionts to plants (Li et al., 2017). Similarly, BEV, *Cardinium*, and *Rickettsia* also infect various tissues in their respective hosts, including salivary glands or stylets (Purcell et al., 1986; Mitsuhashi et al., 2002; Gottlieb et al., 2006; Sacchi et al., 2008; Marubayashi et al., 2014; Gonella et al., 2015). As such, bacteria from these hosts may be inoculated directly into the plant tissue or vascular cells.

Direct access to the cellular milieu may be important for the persistence of these microorganisms, given their intracellular lifestyle. However, intracellular symbionts of insects are known to stay viable and invasive outside of the cells (Purcell et al., 1986; Rasgon et al., 2006). Thus, apart from direct ‘injection’ into the plant tissues, bacteria may also be passed from insect to plant in the form of surface contamination, likely by feces or in honeydew. The intracellular ‘pea aphid *Bemisia*-like symbiont’ (*Hamiltonella defensa*), which can be horizontally transmitted via a bacteria-enriched artificial diet, was detected in some aphid honeydew and siphuncular fluid samples, but was absent from salivary gland secretions (Darby and Douglas, 2003), suggesting transmission via contamination.

The question as to whether intracellular symbionts commonly use surface contamination as a transmission mode could be addressed by artificially inoculating symbionts onto plants and examining whether they can be acquired by uninfected insects. This experiment has already been performed for BEV, which can spread to insects through feeding on contaminated plants, but failed to infect new individuals when artificially sprayed on the surface of the leaves (Purcell et al., 1994). The authors postulated that in this case, the survival of the symbiont outside of the plant was probably poor (Purcell et al., 1994), but other as yet unknown factors could also be necessary to facilitate transmission. Further experiments are required to understand the most efficient ways in which insect symbionts can be shared with plants, which conditions are necessary for the transfers to occur, and how insect-to-plant transfers can be enhanced experimentally.

### Plant–Symbiont Interaction

Endosymbiont survival within or on the surface of the plant is necessary to complete plant-mediated horizontal transmission. Given that these microbes predominantly infect insects and are adapted to the insect intracellular environment, their survival in association with plants is remarkable. Additionally, symbionts entering plant tissues likely need to overcome or resist plant defenses. Plants defend themselves against microbial pathogens and herbivorous insects through the jasmonic acid (JA) and salicylic acid (SA) pathways (Erb et al., 2012; Pieterse et al., 2012). Insect feeding and necrotrophic pathogen attack induce the JA pathway, which leads to the production of repellent, antinutritive, or toxic compounds (Howe and Jander, 2008). The main function of SA pathway is orchestrating the response to biotrophic pathogens. This response involves oxidative bursts, callose deposition, ethylene production, and induction of defensive genes, including antimicrobial proteins (Durrant and Dong, 2004). Both pathways have an antagonistic effect on each other (Pieterse et al., 2012), imposing a trade-off between defense against herbivore attack and concomitant bacterial challenge, which possibly facilitates symbiont transfer from herbivores to plants.

Detection of *Wolbachia* and *Rickettsia* RNA within the tissues of plants confirms that intracellular insect symbionts can survive in this unusual niche (Caspi-Fluger et al., 2012; Li et al., 2017). Moreover, *Wolbachia* was observed to persist in plants for extended periods of time and disseminate to neighboring leaves (Li et al., 2017), which suggests that it can acquire nutrients from plant tissues. Intriguingly, electron microscopy revealed a *Wolbachia*-like bacterium within the vacuole of the phloem cell of the plant (Li et al., 2017). *Wolbachia* has been shown before to be able to invade insect cells in culture (White et al., 2017), but an invasion of a plant cell *in vivo* would likely require different machinery. Whether this is a singular case of intracellular localization or a common *Wolbachia* niche in plants remains to be determined. If the morphological identification of *Wolbachia* within the plant cell is correct, it may represent a biological interaction, with for instance nutrient provisioning of the symbiont in this *Wolbachia*-plant system. Despite the apparently close *Wolbachia*-plant association, *Wolbachia* had no observable effects on the health of *Gossypium hirsutum* (Li et al., 2017). In contrast to *Wolbachia*, BEV did not disseminate in the plant when injected into the phloem by the leafhopper *E. variegatus*, which is surprising given that this bacterium originates from a lineage of plant pathogens (Degnan et al., 2011). Consequently, just like *Wolbachia*, it also did not cause observable pathology in plants (Purcell et al., 1994). The examples above indicate that different symbionts can act differently, sometimes counterintuitively, once they reach the plant.

Overall, we have a poor mechanistic understanding of interactions occurring between plants and intracellular insect-associated bacteria and many questions remain. Do intracellular insect symbionts injected to the plant or their effectors influence plant biology? Do plants mount an immune response against these organisms? And are plants stably or transiently colonized by these bacteria?

The observation that disease causing agents, such as Aster Yellows phytoplasma strain Witches’ Broom, actively alters plant biology and development in a manner that potentiates transmission (MacLean et al., 2014; Orlovskis and Hogenhout, 2016) makes it worth investigating whether symbionts have evolved likewise. In this context, it is notable that insect endosymbiont-derived compounds can affect plants. GroEL, a bacterial chaperonin highly expressed by aphid obligate bacteriocyte-enclosed *Buchnera aphidicola*, was found in aphid saliva and heterologously expressed protein was shown to elicit an immune response in plants (Chaudhary et al., 2014; van Bel and Will, 2016). Further studies in these systems are required to obtain a more thorough understanding of the interactions between plants and insect symbionts.

### Symbiont Acquisition by a New Insect

Similar to symbiont transfer from insect to plant, successful insect acquisition of a symbiont from plant likely depends on a number of insect, symbiont, and plant factors. Location of the symbiont in the plant would appear critical. *Wolbachia* and *Cardinium* (as well as *Arsenophonus*) were found in the phloem of plants which likely increased their chances of being acquired by phloem feeders (Bressan et al., 2012; Gonella et al., 2015; Li et al., 2017). Once in the insect gut, symbionts have to resist digestion, alkaline pH, constitutive reactive oxygen species production, and all the activities of the gut microbiota (Vallet-Gely et al., 2008). Moreover, unless they are able to attach quickly, they could be eliminated by peristaltic gut movements (Vallet-Gely et al., 2008). Subsequently, symbionts have to traverse the peritrophic matrix, invade the gut epithelium or pass through extracellular spaces, enter the body cavity, and localize within appropriate insect tissues. If these bacteria stimulate insect immunity, then avoidance of, or resistance to, the immune responses may also be required. Infection of the appropriate tissue within the germline to ensure vertical transmission is probably not easy to achieve either, with several barriers on the way including the epithelial sheath, peritoneal sheath, and follicular epithelium (Hughes and Rasgon, 2014). Despite these challenges, diverse classes of symbionts have been reported to be vertically transmitted after horizontal transfers, suggesting that symbionts possess innate qualities enabling them to infect germline (Russell and Moran, 2005; Frydman et al., 2006; Weiss et al., 2006; Hughes et al., 2014; Nakayama et al., 2015).

The literature on symbiont acquisition from plants reports various outcomes for the symbiont and insect. In the case of *Rickettsia*, we only know that symbiont DNA has reached the recipient host (Caspi-Fluger et al., 2012), while BEV was shown to be alive in the plant-feeding *E. variegatus* (Purcell et al., 1994). *Cardinium* accumulated in the guts of *M. quadripunctulatus* and *E. vitis* (Gonella et al., 2015), although it is not clear if it established in the insects, as assays were performed immediately after cessation of feeding on the symbiont-contaminated plant. In contrast, *Wolbachia* was reported to be found in the adult progeny derived from the newly infected *B. tabaci* females (Li et al., 2017), suggesting a stable infection.

The examples discussed above add to the body of the literature on insects acquiring their symbionts from a food source (Heath et al., 1999; Darby and Douglas, 2003; Le Clec’h et al., 2013). It is possible that some symbiont–insect host combinations, plant species, and conditions support these horizontal transmission events (Sintupachee et al., 2006; Stahlhut et al., 2010; Morrow et al., 2014; Ahmed et al., 2016). Importantly, the observation that DNA of the symbiont can be acquired from the food source should be taken into account in screens attempting to estimate population infection frequencies from PCR assays on whole insects collected in the field. Symbiont DNA in the insect gut is not equivalent to an infection, and there are cases where orally acquired symbionts failed to establish (Chiel et al., 2009; Faria et al., 2016). Performing screens using dissected tissues different than the gut and carefully avoiding contamination with the gut content may overcome this bias. Confirmation by *in situ* hybridization would provide further evidence of the infection.

### Symbiont Establishment in the Newly Infected Insect Line

Once the symbiont infects the new insect host and establishes efficient vertical transmission, it may induce a cost for its new host. Thus, the symbiont has to prevent elimination from the newly infected lineage by natural selection and only symbionts capable of manipulating their new host biology can persist. Two general strategies facilitate facultative symbiont establishment: providing fitness benefit or manipulating the reproduction (Moran et al., 2008). Furthermore, phenotype induced by the symbiont in the new insect host species may differ from that in the original host (McGraw et al., 2002; Sasaki et al., 2002; Hornett et al., 2008; Le Clec’h et al., 2012).

As closely related insects share similar genetic background and adaptations, at least in some cases intraspecies transfers may be more efficient than interspecies transfers (Łukasik et al., 2015). However, patterns of symbiont distribution in different host species indicate that interspecies transfers occur as well (Tagami and Miura, 2004; Sintupachee et al., 2006; Stahlhut et al., 2010; Morrow et al., 2014; Ahmed et al., 2016). Overall, the efficiency of symbiont establishment in the new lineage upon plant-mediated horizontal transmission depends not only on the symbiont but also on the host’s genetic makeup, physiology, and population structure as well as environmental factors. Even if symbionts rarely succeed in particular transfer events the insect–symbiont–plant interface is a platform for horizontal transmission to occur.

## Ecological and Evolutionary Consequences of Plant-Mediated Horizontal Transfer

Horizontal transmission can have profound consequences for the ecology and evolution of the symbionts and their hosts. It has been postulated that insect symbionts may adapt to utilize plants as a means for horizontal transmission and evolve toward increased virulence within them (Frago et al., 2012). This is likely the case of bacteria in the genus *Arsenophonus*, which have transitioned from being arthropod symbionts to insect-vectored plant pathogens at least twice in their evolutionary history (Bressan et al., 2012). Based on molecular phylogeny and host range, similar conclusions could be drawn for *Spiroplasma*, another widespread clade of arthropod symbionts (Lo et al., 2015). In contrast, some plant pathogens have adapted to live in symbiosis with insects (Degnan et al., 2011; Flórez et al., 2017). Thus, there is an evolutionary continuum in the spectrum of interactions, with some vertically transmitted symbionts of herbivores evolving toward increased plant-mediated transmission and pathogenesis in plants, and some insect-vectored plant pathogens evolving toward efficient vertical transmission in insect populations. In addition to driving adaptation, horizontal transmission broadens a symbiont’s host range. Crossing species and kingdom barriers and establishing symbioses with new hosts can strengthen the selection on parts of the genome that would have otherwise deteriorated and will select to maintain systems required for transfer. Potential coinfections enable symbiont competition with respect to speed of bacterial replication and higher bacterial load within the insect host, and these two are often correlated with higher virulence in the insect host. Moreover, coinfections allow recombination, creating new variants and potentially mitigating against mutational decay through Muller’s ratchet. Horizontal transmission may also drive the evolution and persistence of parasites, such as BEV, as it at least in part unties the fitness of the symbiont from the fitness of the insect host. Moreover, it selects for symbiont genotypes able to survive in the plant and in the new host midgut, and able to invade insect tissues, and proliferate to compensate for stochastic and host-induced symbiont loss on the way to the germline. Together with the relaxed pressure for not harming their hosts, plant-mediated horizontal transmission likely selects for more virulent bacteria.

Finally, horizontal transmission can result in infection of previously uninfected insect lineages and species (Sintupachee et al., 2006; Stahlhut et al., 2010; Morrow et al., 2014), a superinfection with a different symbiont genotype, or a replacement of the original symbiont by the newly acquired one. The consequences of these events for the host have been reviewed elsewhere (Sudakaran et al., 2017). Intriguingly, if horizontal transmission of transovarially transmitted insect symbionts via plants (or any other route) occurs efficiently from both male and female donors, males are not an evolutionary dead end for these organisms (Engelstädter and Hurst, 2009). Transmission through males also implies that the new reproductive manipulations are less likely to be favored (Engelstädter and Hurst, 2009). Therefore, each plant-mediated symbiont transmission event may be a turning point in the history of the symbiont, insect, and plant species.

## Future Endeavors

As we are just starting to explore the cases of plant-mediated horizontal transmission of intracellular bacterial symbionts, there is still much to discover about these systems. Some important questions include: How common are these interactions? How frequently does this form of transmission lead to stable infection in recipient hosts? If the infections are transient, what implications do they have for host biology? And how do insect symbionts influence plants? The broad nature of these questions means that multidisciplinary collaborations would be required to address them.

It would seem imperative to identify how commonly intracellular insect symbionts associate with plants. The existing reports have already provided evidence that at least four different symbionts, five insects, and five plant species could participate in plant-mediated transmission (**Table 1**; Purcell et al., 1994; Caspi-Fluger et al., 2012; Gonella et al., 2015; Li et al., 2017). Screening plant tissues for intracellular symbionts by PCR assay could provide evidence of plant colonization. Another method to identify symbionts in plants would be to screen next generation sequencing (NGS) data for symbiont-derived reads, as many NGS projects aimed at characterizing hosts serendipitously sequenced symbionts too (Salzberg et al., 2005a,b, 2009; Richardson et al., 2012; Gutzwiller et al., 2015). This should be done carefully, to exclude cases of insect contamination and horizontal gene transfer from bacteria to the plant. Similarly, it may be possible to leverage plant RNAseq data to gain insights into the transcriptional activity of symbionts in plants. However, suitable data sets are currently scarce due to the common poly-A enrichment procedure eliminating most of the bacterial transcripts.

Once we have a better understanding of these systems, plant-mediated symbiont transfer could be used for transinfecting new phytophagous insect lineages or species. Moreover, symbiont–plant associations could be exploited from an applied perspective to seed or facilitate the spread of a particular symbiont that induces desirable phenotypes in a herbivorous insect pest. Plant-mediated symbiont transfer may also provide a system suitable to explore the mechanisms and evolutionary trajectories leading to host switching by intracellular insect symbionts and the emergence of insect and insect-vectored plant pathogens.

## Author Contributions

EC and GLH conceived, designed, and wrote the manuscript. GDDH and KP-S provided input and edited the manuscript. EC designed the figure. All authors approved the final version.

## Conflict of Interest Statement

The authors declare that the research was conducted in the absence of any commercial or financial relationships that could be construed as a potential conflict of interest.
